# Epidemiological trends and climatic drivers of pediatric respiratory infections in Wuhan, China: a multi-pathogen analysis

**DOI:** 10.3389/fcimb.2025.1624638

**Published:** 2025-09-04

**Authors:** Changzhen Li, Lei Xi, Jingjing Rao, Feng Tang, Yun Xiang, Xiaomei Wang

**Affiliations:** Department of Laboratory Medicine, Wuhan Children’s Hospital (Wuhan Maternal and Child Healthcare Hospital), Tongji Medical College, Huazhong University of Science & Technology, Wuhan, China

**Keywords:** pediatric respiratory infections, respiratory viruses, mycoplasma pneumoniae, meteorological factors, distributed lag nonlinear models (DLNM), climate-sensitive surveillance

## Abstract

**Objectives:**

To characterize the epidemiology of pediatric respiratory infections and evaluate the lagged, nonlinear associations between meteorological factors and pathogen activity in post-COVID-19 Wuhan, China.

**Methods:**

A total of 28,903 respiratory specimens were collected from pediatric patients at a tertiary hospital between November 2023 and February 2025. Seven pathogens—*Mycoplasma pneumoniae*, adenovirus, respiratory syncytial virus (RSV), influenza A/B, and parainfluenza virus types I/III—were detected using multiplex RT-PCR. Epidemiological patterns were analyzed by age, sex, seasonality, and clinical setting. Daily meteorological data (temperature, relative humidity, wind speed) were aggregated citywide and temporally matched to case data. Spearman correlation and generalized additive models integrated with distributed lag nonlinear models (GAM-DLNMs) were used to assess pathogen-specific climatic sensitivity.

**Results:**

*M. pneumoniae* (18.9%), adenovirus (14.5%), and RSV (9.1%) were the most prevalent pathogens. Distinct age- and sex-specific distributions were observed, with *M. pneumoniae* peaking in school-aged boys and RSV in infants. Seasonal peaks were evident: RSV and influenza A predominated in winter, while *adenovirus* peaked in spring. Meteorological analysis revealed pathogen-specific associations: low humidity preceded RSV surges by 7–14 days; influenza B was strongly associated with wind exposure; and extreme climatic conditions showed heterogeneous effects on transmission risk across pathogens.

**Conclusions:**

This study demonstrates the utility of GAM-DLNMs in capturing climate-sensitive, time-lagged transmission dynamics for multiple pediatric respiratory pathogens. The findings support the development of localized, climate-informed early warning systems to enhance respiratory disease surveillance and preparedness.

## Introduction

Acute respiratory infections (ARIs) remain a leading cause of childhood morbidity and mortality globally, with pneumonia accounting for over 700,000 deaths annually in children under five years of age (Zar and Mulholland, 2003). In China, ARIs continue to drive substantial pediatric clinic visits and hospitalizations (Cui et al., 2024). Although multiple viral and atypical pathogens are implicated, their prevalence varies by age, geography, and season (Hai-Lin et al., 2017). Respiratory syncytial virus (RSV) is a major cause of lower respiratory infections in infants, while *Mycoplasma pneumoniae*(*M. pneumoniae*) is more common in school-aged children (Kumar et al., 2018). Influenza viruses (IFV-A/B), parainfluenza viruses (PIV), and adenovirus (AdV) also contribute significantly to the disease burden (Cai et al., 2014). Regional studies across China have reported substantial variation in pathogen distribution and timing (Liu et al., 2018). Understanding local epidemiology is essential for tailored clinical management and public health planning.

Environmental factors—particularly temperature, humidity, and wind speed—strongly influence respiratory virus activity (Wenfang et al., 2020). Numerous studies have shown that low temperatures and specific humidity conditions can enhance viral transmission, particularly for influenza and RSV (Baker et al., 2019; Yin et al., 2023). However, such associations are often non-linear and delayed, necessitating advanced time-series methods to fully capture their effects. In the post-COVID-19 era, widespread non-pharmaceutical interventions (NPIs) led to a temporary suppression of common respiratory viruses, creating an “immunity debt” among children (Terliesner et al., 2022). Following the relaxation of NPIs, many regions—including China—have experienced out-of-season surges in RSV, *M. pneumoniae*, and AdV, likely driven by this immunity gap (Del Riccio et al., 2024). These changing patterns call for renewed surveillance using robust analytical approaches. Despite the recognized impact of weather on respiratory infections, few studies have examined multiple pediatric pathogens over a prolonged post-pandemic period while accounting for environmental factors. Wuhan, a megacity in central China with a subtropical monsoon climate, high population density, and historical significance in the COVID-19 pandemic, provides an ideal setting to investigate the interplay between environmental factors and respiratory pathogen dynamics in the post-pandemic context.

In this study, we aimed to characterize the epidemiological and environmental patterns of seven key pediatric respiratory pathogens (*M. pneumoniae*, AdV, RSV, IFV-A/B, PIV-I/III) in Wuhan from 2023 to 2025. Using generalized additive models (GAMs) integrated with distributed lag non-linear models (DLNMs), we explored the non-linear and lagged effects of temperature, humidity, and wind speed on pathogen positivity rates. These findings may inform climate-based early warning systems and guide targeted interventions to mitigate pediatric respiratory disease burden.

## Materials and methods

### Study population and data collection

This retrospective study was conducted at a tertiary pediatric hospital in Wuhan, China, between November 2023 and February 2025. Pediatric patients aged 0 to 18 years who presented to the outpatient or inpatient departments with acute respiratory symptoms were consecutively enrolled. An acute respiratory infection was defined as the presence of at least one of the following clinical features: cough, fever (axillary temperature ≥38 °C), sore throat, nasal congestion, or dyspnea. Eligible patients were those with clinical diagnoses of acute respiratory tract infections who provided respiratory specimens (throat swabs) for laboratory testing. Patients were excluded if they had underlying chronic respiratory diseases (e.g., asthma, bronchopulmonary dysplasia), known immunosuppressive conditions (e.g., malignancy or immunodeficiency), incomplete clinical or demographic data, or declined to participate. After applying these criteria, a total of 28,903 pediatric cases were included in the final analysis. All data were anonymized prior to analysis, and the study protocol was approved by the Medical Ethics Committee of Wuhan Children’s Hospital.

### Methods for the detection of seven respiratory pathogens

Respiratory specimens, including sterile throat swabs were collected following standardized clinical protocols. All samples were immediately placed into viral transport medium (UTM™, Copan Diagnostics, Murrieta, CA, USA) and transported within 2 hours at 2-8 °C to the clinical laboratory. Nucleic acid extraction was performed using a standardized automated extraction platform following the manufacturer’s instructions. Multiplex real-time polymerase chain reaction (RT-PCR) assays were conducted for the simultaneous detection of ADV, RSV, IFV-A, IFV-B, and PIV-I, PIV-III using a commercially available Respiratory Virus Nucleic Acid Six-Plex PCR Kit (real-time fluorescence PCR) (Zhuocheng Huisheng Biotechnology Co., Ltd., Beijing, China). Additionally, detection of *M. pneumoniae* was performed using the *M. pneumoniae* Nucleic Acid Detection Kit (fluorescence PCR method) (Baoruiyuan Biotechnology Co., Ltd., Beijing, China). PCR amplification and fluorescence detection were conducted on an ABI 7500 Real-Time PCR System (Applied Biosystems, Thermo Fisher Scientific, Foster City, CA, USA), according to the respective manufacturers’ protocols. The seven pathogens — ADV, RSV, IFV-A, IFV-B and PIV-I, PIV-III — were selected due to their high clinical relevance and burden in children. These viral and atypical pathogens are the main causes of paediatric acute respiratory infections and are routinely detected in our hospital using multiplex RT-PCR. Bacterial and fungal pathogens were excluded as throat swabs are only suitable for accurate detection to a limited extent, there is a risk of colonisation or contamination and there are no standardised high-throughput diagnostics. In addition, viral and atypical pathogens are more sensitive to climatic influences, which corresponds to the environmental focus of this study.

### Climatic data collection

In Wuhan, the city is divided into 13 administrative districts, each containing a total of 176 environmental monitoring stations. Since the majority of samples from hospitalized patients were collected from these districts, we utilized averaged data from all monitoring stations as the city-wide environmental indicators, with outliers excluded based on Grubbs’ test. Meteorological parameters, including mean temperature (°C), relative humidity (%), and wind speed (km/h), were obtained from the Wuhan Meteorological Bureau’ s network of automated weather stations. Air pollutant levels, specifically fine particulate matter (PM2.5, μg/m³), carbon monoxide (CO, mg/m³), nitrogen dioxide (NO2, μg/m³), and ozone (O3, μg/m³), were sourced from the China National Environmental Monitoring Center (CNEMC). These environmental data were aggregated daily and spatially averaged across monitoring sites to reflect city-wide exposure, then temporally aligned with pathogen detection outcomes to enable time-series analyses.

### Statistical analysis

Statistical analyses were performed to characterize the epidemiology of seven respiratory pathogens and explore their associations with environmental factors. Descriptive statistics were used to calculate the overall positivity rates (%) for *M. pneumoniae*, ADV, RSV, IFV-A, IFV-B, PIV-I, and PIV-III, stratified by age group (<1, 1-3, 3-6, 6-11, >11 years), and gender. Differences across strata were evaluated using chi-square tests, with Bonferroni correction applied to adjust for multiple comparisons. Spearman correlation analysis was conducted to examine the monotonic relationships between the positivity rates of major respiratory pathogens and meteorological variables (relative humidity, temperature, and wind speed). Pairwise correlations were calculated using Spearman’s rank correlation coefficient (r_s_), with corresponding P-values. Correlation matrices were visualized using a pair plot combining histograms, scatterplots with LOESS smoothing, and annotated correlation coefficients.

To further investigate lagged and non-linear effects of Meteorological exposures, DLNMs were integrated with the GAM framework. Cross-basis functions were constructed using natural cubic splines for both exposure and lag dimensions (0–21 days) (Zhang et al., 2024), with the DLNM formulated as:


$$\begin{array}{l} log\left(E\left(Y_{t}\right)\right)=\alpha +cb\left(Meteorological\;variables,df,\;lag\;=\;0-21\right)\\ \;+\text{ns}\left(\text{Pollutantt},\text{df}\right)\;+\text{ ns}\left(\text{Time},\text{ df}\right)\;+\text{ factor}\left(\text{DOW}\right)\;\\ \;+\text{ factor}\left(\text{Holiday}\right)+log\left(Total\;samples_{t}\right) \end{array}$$

where *Y_t_* represents daily positive cases of each pathogen, α is the intercept of the model; cb(*Meteorological variables*) represents the cross-basis function capturing exposure-lag-response relationships, including average temperature, relative humidity and wind speed. Natural cubic spline functions (ns) were employed to flexibly model nonlinear relationships. Meteorological variables, including temperature, relative humidity, and wind speed, were incorporated using DLNMs to capture both lagged and nonlinear effects. The degrees of freedom (df) for the exposure-lag structures were selected based on the Akaike Information Criterion (AIC), ensuring optimal model fit. Air pollutants (PM2.5, PM10, SO_2_, NO_2_, CO, and O_3_) were initially considered as potential confounders. However, due to multicollinearity among certain pollutants—specifically, a variance inflation factor (VIF) >5 between PM10 and several other variables—PM10 was excluded from the final model. The remaining pollutants (PM2.5, SO_2_, NO_2_, CO, and O_3_) were incorporated as covariates using natural cubic splines with 3 degrees of freedom to flexibly adjust for their nonlinear confounding effects (Wang et al., 2022). A natural spline function of time with 7 df per year was used to control for long-term trends. Additionally, categorical variables including day of the week (DOW), and holiday (Holiday) were included to adjust for short-term calendar-related variations. The log-transformed total number of daily tested samples was incorporated as an offset to account for variations in the sampling effort. Sensitivity analyses were performed by varying the degrees of freedom for spline functions and substituting alternative pollutant variables to assess the robustness of model estimates. Model diagnostics confirmed the validity of the GAM + DLNM models. Pearson residual-based plots (residual-vs-fitted and Q–Q plots) showed no major violations of distributional assumptions or variance homogeneity. All analyses were performed using R (version 4.x), with statistical significance set at two-sided *P* < 0.05.

## Results

### Overview of pathogen prevalence and the landscape of co-infections

**Figure 1:**
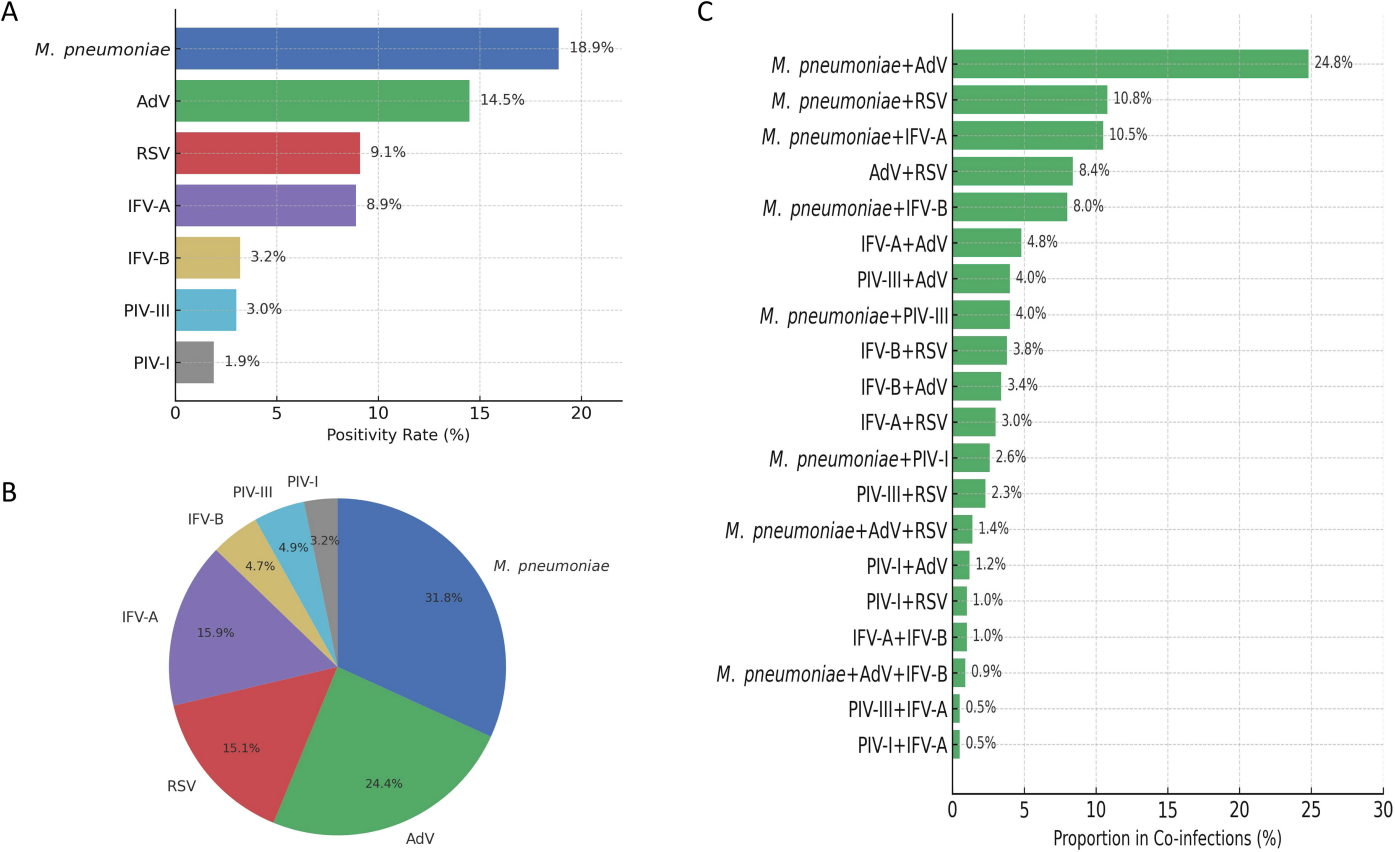
Overview of pathogen detection and co-infection patterns among pediatric respiratory specimens. **(A)** Positivity rates for seven respiratory pathogens detected among 28,903 specimens collected between November 2023 and February 2025. *M. pneumoniae* (18.9%), ADV (14.5%), and RSV (9.1%) were the most frequently identified. **(B)** Distribution of single infections, with *M. pneumoniae* accounting for 31.8% of all mono-pathogen cases. **(C)** Leading co-infection combinations, predominantly involving *M. pneumoniae* and ADV (24.8%), followed by *M. pneumoniae* with RSV (10.8%) and IFV-A (10.5%).

Of the 28,903 respiratory specimens tested between November 2023 and February 2025, 54.1% (n = 15,638) were positive for at least one of seven pathogens, with *M. pneumoniae* being the most common (5,449 cases, 18.9%), followed by AdV (4,191 cases, 14.5%) and RSV (2,618 cases, 9.1%) (**Figure 1A**). Single infections accounted for 49.1% of all cases and were predominantly *M. pneumoniae* (4,507 cases, 31.8%), AdV (3,458 cases, 24.4%) and IFV-A (2,256 cases, 15.9%) (**Figure 1B**). Co-infections were predominantly combinations around *M. pneumoniae*, especially with AdV (361 cases, 24.8% of co-infections), RSV (158 cases, 10.8%) and IFV-A (153 cases, 10.5%). Other notable combinations were AdV + RSV (122 cases, 8.4%) and *M. pneumoniae* + IFV-B (117 cases, 8.0%) (**Figure 1C**), indicating possible pathogen synergies and seasonal overlap.

### Demographic variations in pathogen distribution and co-infection patterns

**Figure 2:**
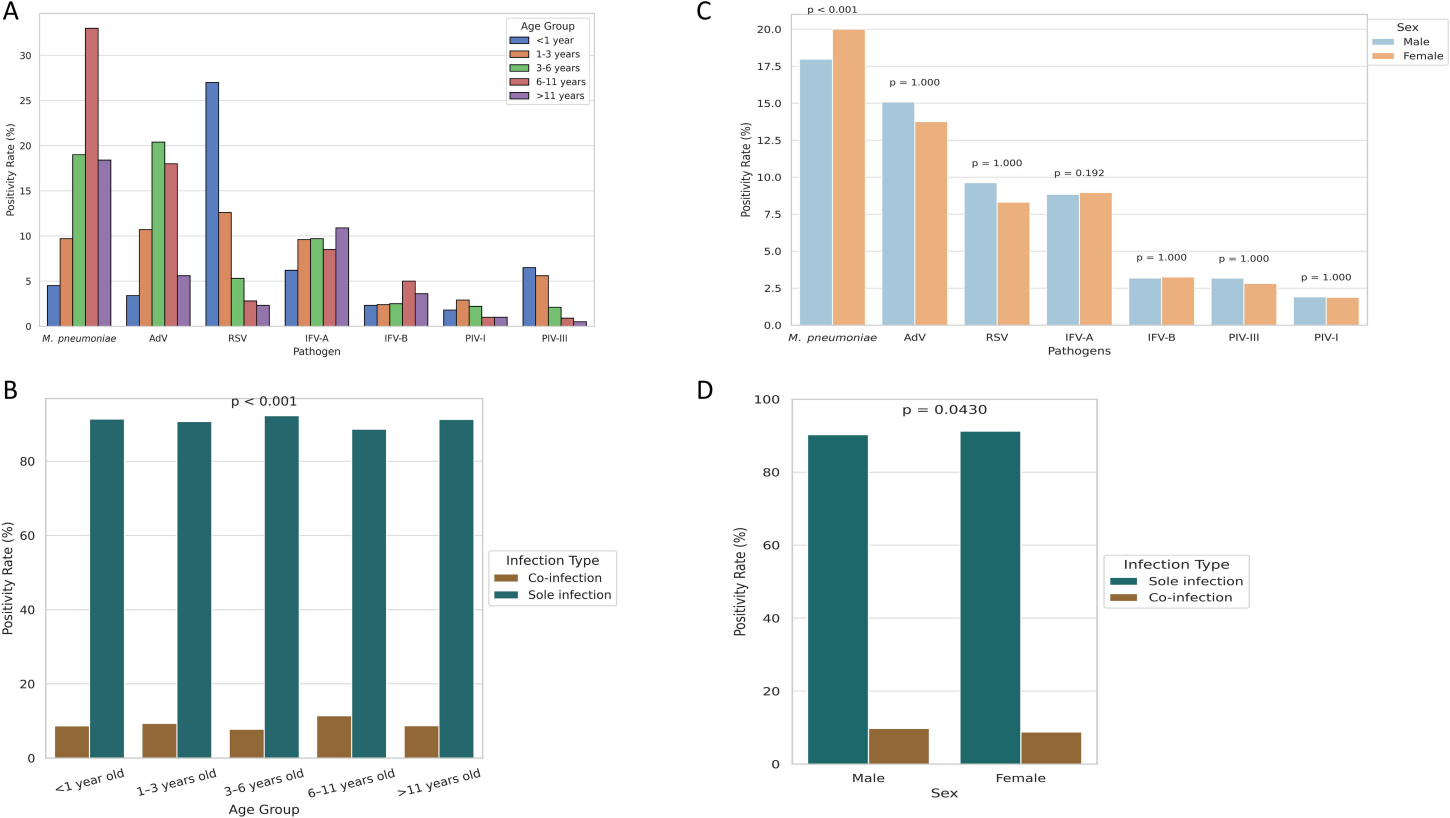
Age- and sex-specific differences in pathogen positivity and co-infection rates.**(A)** Age-stratified positivity rates reveal distinct infection profiles across respiratory pathogens. **(B)** Co-infection rates by age group, highlighting higher prevalence in school-aged children. **(C)** Sex-based differences in positivity, with statistical significance observed only for *M. pneumoniae*. **(D)** Co-infection rates stratified by sex, showing a slightly higher rate in females.

Age-specific analysis revealed distinct epidemiologic profiles (**Table 1**; **Figure 2A**). *M. pneumoniae* positivity increased with age, peaking at 33.0% in the 6 – 11-year group and declining in adolescents (*P* < 0.001). AdV peaked earlier, with highest positivity (20.4%) in children aged 3 – 6 years (*P* < 0.001). In contrast, RSV was most common in infants (27.0%) and declined steadily with age, reaching 2.3% in adolescents. IFV-A showed a gradual increase with age, peaking at 10.9% in adolescents (*P* = 0.018). IFV-B and PIV-I remained low (1 – 5%) across all age groups. PIV-III was most prevalent in infants and toddlers (6.5% and 5.6%, respectively), with rates falling below 1.0% after age 6, indicating early-life susceptibility. Co-infection patterns also varied by age (*P* < 0.001; **Figure 2B**), with the highest rates in the 6 – 11-year group (11.4%), followed by the 1 – 3-year (9.3%) and <1-year (8.6%) groups, and lowest in the 3 – 6-year group (7.7%). Among the seven pathogens, only *M. pneumoniae* showed a significant sex difference, with higher positivity in males (20.0%) than females (17.9%) (*P* < 0.001; **Figure 2C**). No consistent sex differences were observed for other pathogens. Coinfection rates were slightly higher in females (6.9%) than in males (6.6%) (*P* = 0.043), but the difference was minimal (**Figure 2D**).

**Table 1:** Age-specific distribution of respiratory pathogens among pediatric patients.

Age group (years)	M. pneumoniae, n (%)	AdV, n (%)	RSV, n (%)	IFV-A, n (%)	IFV-B, n (%)	PIV-I, n (%)	PIV-III, n (%)
< 1	188/4223(4.5)	142/4223(3.4)	1141/4223(27.0)	262/4223(6.2)	98/4223(2.3)	74/4223(1.8)	276/4223(6.5)
1-3	553/5723(9.7)	613/5723(10.7)	722/5723(12.6)	549/5723(9.6)	139/5723(2.4)	168/5723(2.9)	322/5723(5.6)
3-6	1781/9358(19.0)	1912/9358(20.4)	497/9358(5.3)	907/9358(9.7)	237/9358(2.5)	209/9358(2.2)	196/9358(2.1)
6-11	2627/7967(33.0)	1433/7967(18.0)	220/7967(2.8)	674/7967(8.5)	396/7967(5.0)	78/7967(1.0)	69/7967(0.9)
> 11	300/1632(18.4)	91/1632(5.6)	38/1632(2.3)	178/1632(10.9)	58/1632(3.6)	17/1632(1.0)	8/1632(0.5)

### Inpatients exhibit higher co-infection rates and distinct pathogen profiles

**Figure 3:**
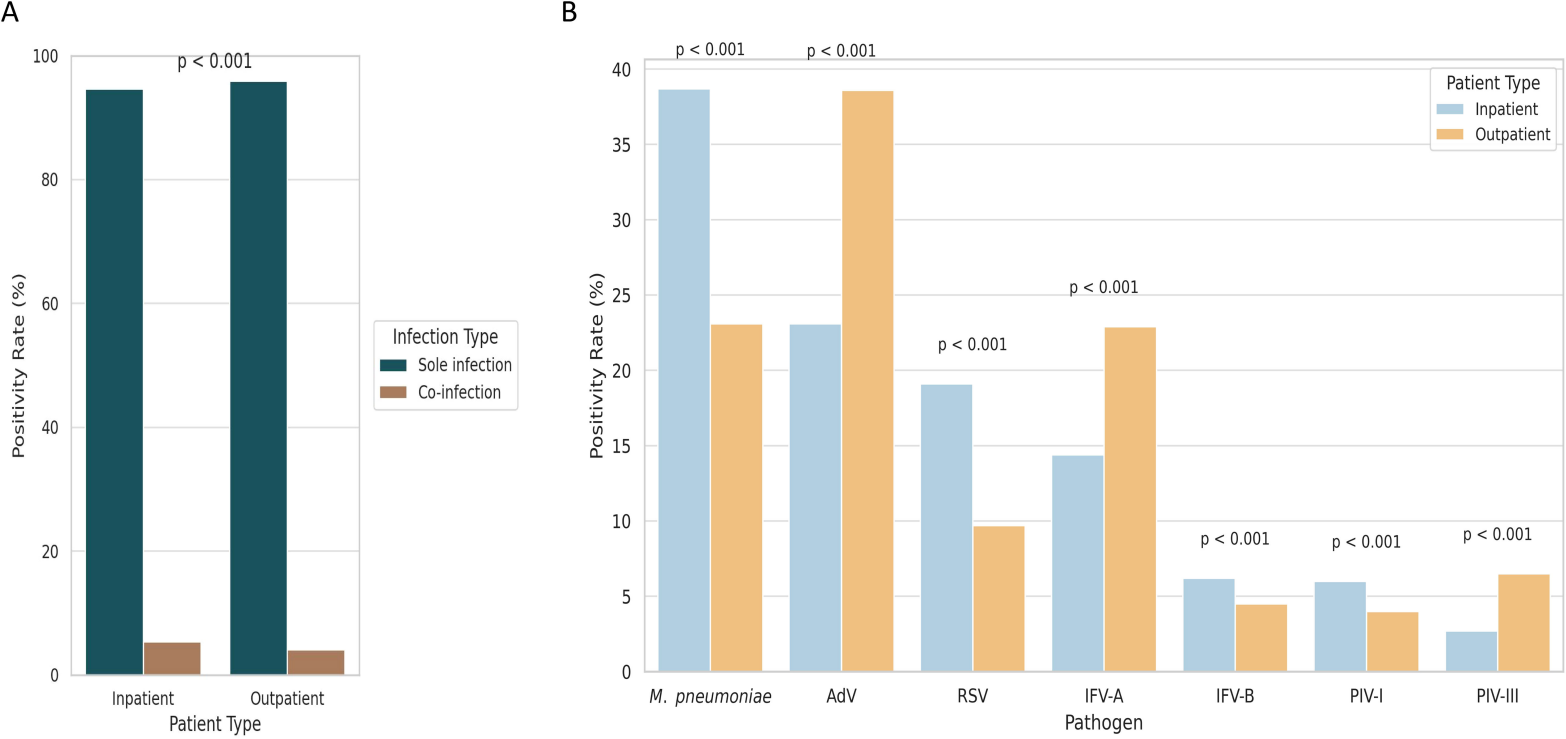
Comparison of pathogen detection and co-infection rates between hospitalized and outpatient pediatric patients. **(A)** Co-infection was more frequently observed in hospitalized children than in outpatients. **(B)** Pathogen-specific positivity rates varied between inpatient and outpatient groups, reflecting differences in clinical severity and diagnostic settings.

Among 28,903 pediatric patients, co-infections were more common in inpatients (5.4%) than outpatients (4.1%, *P* < 0.001; **Figure 3A**). Pathogen distribution also differed significantly between groups (*P* < 0.001; **Figure 3B**): *M. pneumoniae*, RSV, IFV-B, and PIV-III were more prevalent in inpatients, whereas AdV, IFV-A, and PIV-I were more common in outpatients. These findings suggest pathogen-specific patterns linked to clinical setting, potentially reflecting different severity profiles.

### Dynamic seasonal signatures suggest divergent climatic dependencies

**Figure 4:**
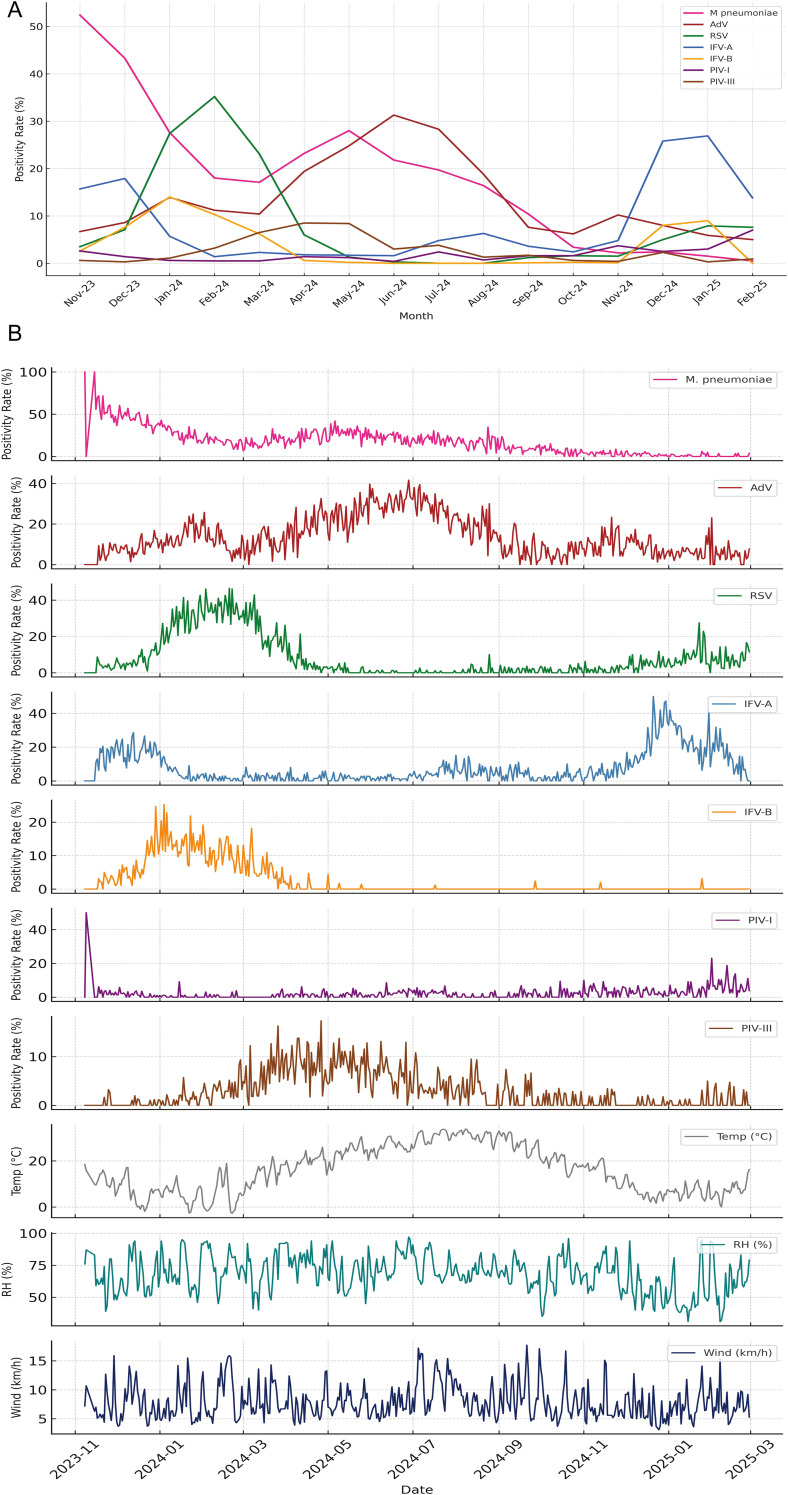
Seasonal dynamics of pediatric respiratory pathogens and associated meteorological patterns. **(A)** Monthly positivity rates of seven respiratory pathogens from November 2023 to February 2025, illustrating distinct seasonal peaks. RSV and IFV-A exhibited pronounced winter surges, while *M. pneumoniae* dominated early in the surveillance period before declining. AdV peaked during late spring to summer, whereas IFV-B, PIV-I, and PIV-III showed persistently low detection without clear seasonal trends. **(B)** Daily positivity rates for the same pathogens plotted alongside three key meteorological indicators—temperature (°C), relative humidity (%), and wind speed (km/h)—revealing short-term fluctuations and potential climate-pathogen interactions. The combined display highlights temporal heterogeneity in pathogen activity and suggests divergent ecological or environmental drivers underlying respiratory infection patterns.

Temporal patterns in both monthly (**Figure 4A**) and daily (**Figure 4B**) positivity rates revealed distinct seasonal dynamics across the seven pediatric respiratory pathogens. RSV and IFV-A exhibited pronounced winter peaks, with RSV reaching its maximum in January–February 2024 and IFV-A showing bimodal surges in December 2023 and again from November 2024 to January 2025. In contrast, *M. pneumoniae* predominated early in the surveillance period (November–December 2023), while AdV demonstrated broader circulation, peaking from late spring to mid-summer. IFV-B, PIV-I, and PIV-III maintained low-level activity throughout the year, with no consistent seasonal peaks. **Figure 4A** illustrates these trends using smoothed monthly positivity rates, facilitating comparisons of temporal dominance and inter-pathogen variation. **Figure 4B** provides daily positivity rates alongside three key meteorological indicators—temperature, relative humidity, and wind speed—offering high-resolution insights into short-term fluctuations and potential lagged climate effects. Together, these findings highlight substantial temporal heterogeneity and suggest that pathogen-specific ecological or behavioral factors, beyond temperature alone, contribute to seasonal transmission patterns. The integration of daily and monthly analyses enhances the resolution of climate–pathogen relationships and provides a foundation for developing seasonally adaptive early warning systems and targeted interventions.

### Meteorological correlations support climate-sensitive transmission

**Figure 5:**
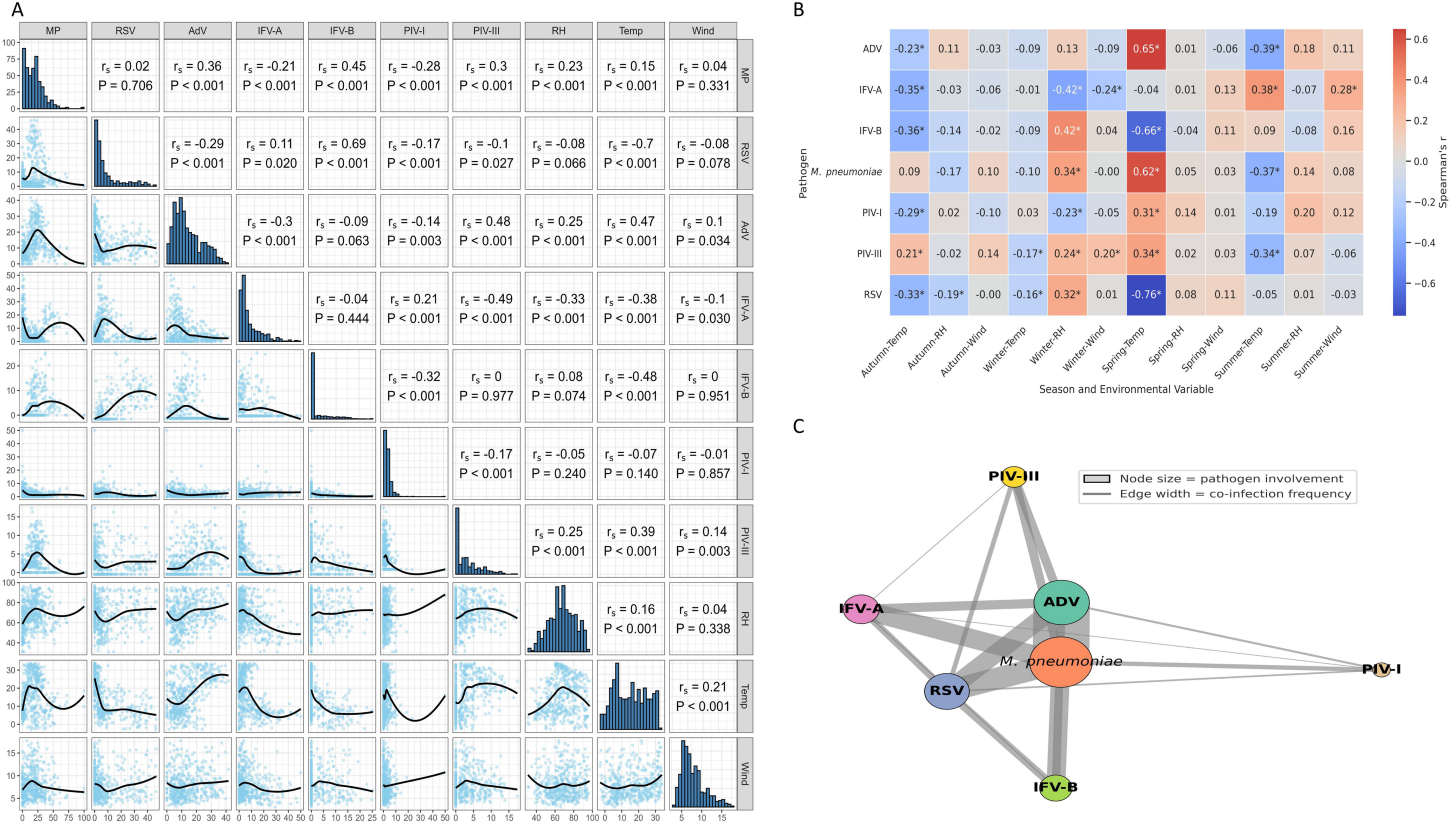
Environmental correlations and co-infection patterns of respiratory pathogens. **(A)** Spearman correlations between pathogen positivity rates and meteorological variables. Notable associations include a negative correlation between RSV and temperature, and positive correlations of *M. pneumoniae* and AdV with both temperature and humidity. **(B)** Season-stratified correlation analysis reveals temporal variation in climate–pathogen relationships, with stronger associations observed during winter and spring. * indicates p < 0.05 (Spearman’s correlation). **(C)** Co-infection network illustrating pathogen co-occurrence. Node size indicates the frequency of involvement; edge width reflects co-infection frequency. *M. pneumoniae* and AdV were the most frequently co-detected pathogens.

Spearman correlation analysis revealed significant associations between pathogen activity and meteorological variables (**Figure 5A**). *M. pneumoniae* was positively correlated with relative humidity (r_s_ = 0.42, *P* < 0.001), while RSV showed a strong inverse correlation with temperature (r_s_ = –0.70, *P* < 0.001). AdV was associated with both higher humidity (r_s_ = 0.36) and temperature (r_s_ = 0.47), whereas IFV-A showed negative correlations with both (r_s_ = –0.44 and –0.38, respectively). IFV-B, PIV-I, and PIV-III had weak or inconsistent associations, suggesting more complex or indirect influences. Seasonal stratification further clarified these patterns (**Figure 5B**): *M. pneumoniae* and AdV correlations with humidity and temperature were strongest in spring, while RSV–temperature correlation peaked in winter (r_s_ = –0.76). IFV-B was most sensitive to cold in spring (r_s_ = –0.66). These results indicate that climate effects on pathogen transmission are both pathogen-specific and season-dependent. A co-infection network analysis (**Figure 5C**) showed that *M. pneumoniae* and AdV were the most interconnected pathogens, frequently co-occurring with RSV, IFV-A, and IFV-B. This suggests potential synergistic interactions or overlapping seasonal windows.

### DLNM models reveal distinct lagged and nonlinear climate effects

**Figure 6:**
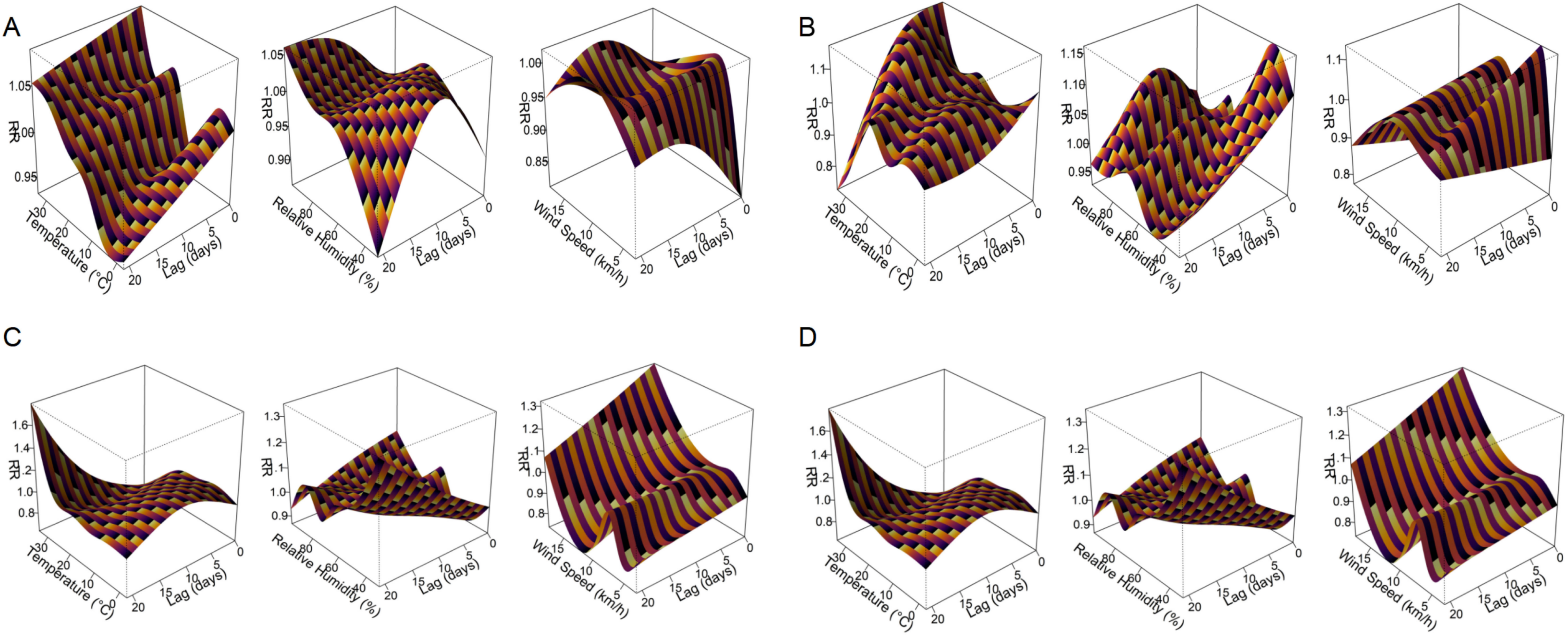
Three-dimensional exposure–lag–response surfaces showing the effects of meteorological factors on respiratory pathogen detection risk. Panels **(A–D)** present the DLNM results for *M. pneumoniae*, AdV, RSV, and IFV-A, respectively. Each response surface depicts the RR of pathogen positivity across a 21-day lag period in relation to temperature, relative humidity, or wind speed. The plots highlight distinct nonlinear and lag-dependent associations between meteorological exposures and respiratory pathogen activity.

The DLNMs showed three predominant types of meteorological response patterns among respiratory pathogens: immediate effects (lag 0 – 2 days), delayed effects (lag 15 – 21 days) and wind-induced susceptibility. These effects varied considerably between pathogens and meteorological variables, with both exposure intensity and lag pattern influencing infection risk (**Figure 6**, **Supplementary Figure S1**). Immediate effects were observed for *M. pneumoniae* and IFV-A under extreme heat (33.8 °C, lag 0), with relative risks (RRs) of 1.09 (95% CI: 1.01 – 1.17) and 1.15 (95% CI: 1.06 – 1.25), respectively. AdV also showed a prompt response to meteorological stressors, with a peak RR of 1.17 (95% CI: 1.08 – 1.27) at lag 2 at high temperature and 1.16 (95% CI: 1.05 – 1.28) at lag 0 at low humidity (44.3%). In addition, PIV-I was highly sensitive to elevated temperature and humidity at lag 0 (RRs of 1.26 and 1.24, respectively; **Supplementary Figure S1**), indicating a rapid transmission potential under warm and humid conditions. The delayed effects were most pronounced for RSV, which showed a peak RR of 1.61 (95% CI: 1.22 – 2.12) at lag 21 after exposure to high temperature (33.8 °C) and 1.13 (95% CI: 1.00 – 1.28) at lag 17 under low humidity (35.6%). Similarly, PIV-III showed a delayed increase in infection risk at lag 21 under dry conditions (31% humidity, RR = 1.49; 95% CI: 1.08 – 2.05), indicating a persistent climatic influence over time. Wind-induced susceptibility varied between pathogens. IFV-B showed the strongest wind-induced association, with a peak RR of 2.47 (95% CI: 1.21 – 5.04) at lag 15 under increased wind speed (17.7 km/h; **Supplementary Figure S1**). RSV and IFV-A also showed moderate responses to wind at shorter delays, with peak RRs of 1.32 and 1.19, respectively.

These pathogen-specific and delay-dependent climate associations are shown in **Figure 6** for the four most common pathogens (*M. pneumoniae*, AdV, RSV and IFV-A), while additional results for IFV-B, PIV-I and PIV-III are shown in **Supplementary Figure S1**. A full summary of peak RR values, 95% confidence intervals and corresponding lag days can be found in **Table 2**, which provides a comprehensive overview of the meteorological impact on all seven pathogens. These results emphasise the value of DLNMs in capturing non-linear and time-dependent environmental risks that would be overlooked by conventional models. The ability to distinguish between immediate and delayed responses provides important insights for the timing of public health measures and the development of climate-informed early warning systems.

**Table 2:** Peak RR, 95% CI, and lag days for meteorological exposures across respiratory pathogens.

Pathogen	Meteorological Variable	Peak Exposure	Lag (days)	Peak RR(95% CI)
*M. pneumoniae*	Temperature	33.8 °C	0	1.09(1.01-1.17)
*M. pneumoniae*	Humidity	97%	18	1.06(1.00-1.12)
*M. pneumoniae*	Wind speed	6.1 km/h	11	1.01(1.00-1.02)
AdV	Temperature	33.8 °C	2	1.17(1.08-1.27)
AdV	Humidity	44.30%	0	1.16(1.05-1.28)
AdV	Wind speed	5.4 km/h	0	1.14(1.06-1.21)
RSV	Temperature	33.8 °C	21	1.61(1.22-2.12)
RSV	Humidity	35.60%	17	1.13(1.00-1.28)
RSV	Wind speed	17.7 km/h	0	1.32(1.05-1.66)
IFV-A	Temperature	33.8 °C	0	1.15(1.06-1.25)
IFV-A	Humidity	84%	12	1.27(1.14-1.40)
IFV-A	Wind speed	3.8 km/h	15	1.19(1.11-1.28)
IFV-B	Temperature	-2.7°C	21	1.7(1.31-2.21)
IFV-B	Wind speed	17.7 km/h	15	2.47(1.21-5.04)
PIV-I	Temperature	27.4 °C	0	1.26(1.08-1.47)
PIV-I	Humidity	97%	0	1.24(1.02-1.50)
PIV-III	Temperature	9.7 °C	17	1.03(1.00-1.07)
PIV-III	Humidity	31%	21	1.49(1.08-2.05)

RR, relative risk; CI, confidence interval. Peak exposure indicates the meteorological condition associated with the highest estimated risk. Results were obtained using distributed lag nonlinear models (DLNM) over a 21-day lag period.

### Extreme quantile effects highlight climate-specific risk profiles

**Figure 7:**
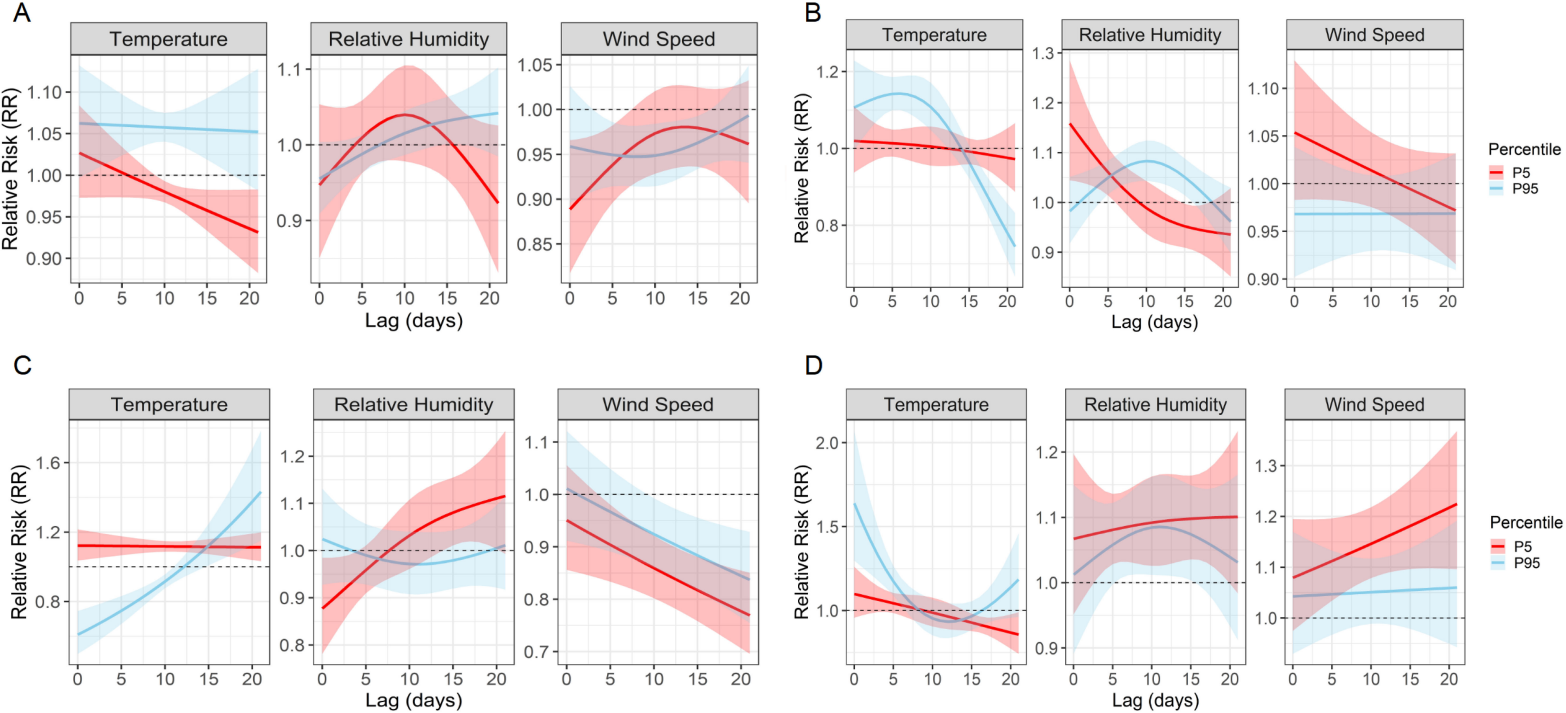
Quantile-based lag–response associations between extreme meteorological exposures and pathogen-specific infection risks. Line plots show the estimated RRs and 95% confidence intervals associated with the 5th (P5, red) and 95th (P95, blue) percentiles of temperature, relative humidity, and wind speed over lag periods of 0–21 days. **(A–D)** correspond to *M. pneumoniae*, AdV, RSV, and IFV-A, respectively. These plots reveal distinct temporal risk profiles and underscore the heterogeneous, pathogen-specific sensitivities to climatic extremes.

Quantile-based DLNMs evaluating the 5th and 95th percentiles of meteorological exposure showed different pathogen-specific sensitivities to climatic extremes (**Figure 7**; **Supplementary Figure S2**). Among the four most common pathogens, *M. pneumoniae* showed moderate susceptibility to extreme heat, with a peak RR of 1.06 (95% CI: 1.00 – 1.12) at lag 1 and a delayed response to high humidity at lag 17 (RR = 1.04, 95% CI: 1.00 – 1.07). AdV showed an increased risk at both low (lag 0, RR = 1.16, 95% CI: 1.04 – 1.29) and high humidity (lag 10, RR = 1.08, 95% CI: 1.04 – 1.12) and responded strongly to high temperature at lag 6 (RR = 1.14, 95% CI: 1.10 – 1.19). RSV remained highly sensitive to extreme weather conditions, with a significant increase in risk at high temperature (lag 21, RR = 1.43), low temperature (lag 0, RR = 1.12) and low humidity (lag 20, RR = 1.11). IFV-A showed the strongest heat-related effect, peaking at lag 0 with RR = 1.64, and showed additional risks from low humidity and low wind speed during longer delays. Less common pathogens also showed remarkable correlations. IFV-B was particularly sensitive to extreme cold (lag 21, RR = 1.56) and high wind speed (lag 15, RR = 2.47). PIV-I responded directly to extreme heat and high humidity (RR = 1.21 and 1.17), while PIV-III was primarily influenced by persistently low humidity (lag 21, RR = 1.21). **Figure 7** shows the RR curves for *M. pneumoniae*, AdV, RSV and IFV-A, while **Supplementary Figure S2** shows the corresponding results for IFV-B, PIV-I and PIV-III. Detailed peak RR values and confidence intervals can be found in **Table 3**. These results highlight the heterogeneous climate sensitivity of respiratory pathogens and illustrate the value of modelling extreme events to inform climate-resilient surveillance systems.

**Table 3:** Peak RR, 95% CI, and lag days for extreme meteorological exposures by pathogen.

Pathogen	Variable	Percentile	Lag	Peak RR (95% CI)
*M. pneumoniae*	Relative Humidity	P95	17	1.04(1.00-1.07)
*M. pneumoniae*	Temperature	P95	1	1.06(1.02-1.12)
ADV	Relative Humidity	P5	0	1.16(1.05-1.29)
ADV	Relative Humidity	P95	10	1.08(1.04-1.12)
ADV	Temperature	P95	6	1.14(1.10-1.19)
RSV	Relative Humidity	P5	20	1.11(1.00-1.23)
RSV	Temperature	P5	0	1.12(1.04-1.22)
RSV	Temperature	P95	21	1.43(1.15-1.78)
IFV-A	Relative Humidity	P5	19	1.10(1.01-1.20)
IFV-A	Relative Humidity	P95	11	1.09(1.01-1.17)
IFV-A	Temperature	P95	0	1.64(1.30-2.07)
IFV-A	Wind Speed	P5	21	1.23(1.10-1.37)
IFV-B	Temperature	P5	21	1.56(1.26-1.95)
PIV-I	Relative Humidity	P95	0	1.17(1.02-1.33)
PIV-I	Temperature	P95	0	1.21(1.01-1.45)
PIV-III	Relative Humidity	P5	21	1.21(1.04-1.41)

RR, relative risk. CI, confidence interval. Values are extracted at the lag with the highest RR under significant conditions (lower CI > 1), for each pathogen-variable-percentile combination.

### Model diagnostics support analytical robustness

Model diagnostics confirmed the robustness of the GAM + DLNM framework. Pearson residual plots and Q–Q plots showed good adherence to model assumptions, including distributional adequacy and variance homogeneity (**Supplementary Figure S3**). The negative binomial model appropriately handled overdispersion. The IFV-B model, however, should be interpreted with caution due to a high proportion (69%) of zero-positive days. Overall, residual patterns support the validity of climate–pathogen associations. Additionally, sensitivity analyses using alternative degrees of freedom and air pollutant variables yielded consistent results, reinforcing model stability.

## Discussion

Our large-scale epidemiological study, conducted from November 2023 to February 2025 in Wuhan, China, systematically characterised the prevalence, demographic patterns, co-infection profiles and meteorological associations of seven major respiratory pathogens in paediatric patients. *M. pneumoniae*, AdV and RSV emerged as the dominant pathogens, with age-stratified analyses revealing different susceptibility windows and meteorological analyses using Spearman correlation and DLNMs identifying climate-sensitive patterns. In particular, pathogen-specific infection risks were modulated by delayed weather exposures and extreme climatic conditions (e.g. 5th and 95th percentiles), emphasising the potential for integrating climate metrics into early warning systems for respiratory diseases.

These findings extend our previous work documenting a significant outbreak of *M. pneumoniae* in Wuhan from October 2022 to October 2023, mainly affecting school-aged children with high co-infection rates with AdV and influenza viruses (Li et al., 2025b). In the post-outbreak period, the prevalence of *M. pneumoniae* declined, consistent with the global pattern of a temporary increase following the COVID outbreak, which peaked in late 2023 and disappeared again in early 2024 (Bolluyt et al., 2024; Centers for Disease Control and Prevention, 2024; Gong et al., 2024; Meyer Sauteur et al., 2024; Nordholm et al., 2024). This trend likely reflects the relaxation of non-pharmaceutical interventions (NPIs) and the accumulation of a population-level immunity debt due to reduced exposure in 2020 – 2022, followed by the re-emergence of seasonal viruses such as AdV, RSV and IFV-A.

The age-stratified patterns in our study confirmed well-established, pathogen-specific epidemiological trends. *M. pneumoniae* infections peaked in school-aged children (6 – 11 years), consistent with increased transmission in crowded educational settings and previous multicentre reports from China (Xu et al., 2024; Zhang et al., 2025). AdV predominates in preschool children (3 – 6 years), probably due to increased exposure in day care centres and immature mucosal immunity, which is confirmed by frequent outbreaks of AdV in day care centres (Li et al., 2025a). RSV mainly affected infants (<1 year), which is consistent with its major role in bronchiolitis and viral pneumonia (Ye and Wang, 2022; Gentile et al., 2025). School-age children in particular showed the highest susceptibility to co-infection, especially with *M. pneumoniae*, confirming previous findings that this pathogen can exacerbate the severity of the disease by interfering with the host’s immune responses and exacerbating respiratory inflammation (Waites et al., 2017; Sondergaard et al., 2018). Co-infections were more frequent and more diverse in hospitalised children than in outpatients, further supporting the link between pathogen multiplication and poorer clinical outcomes (Cilla et al., 2008; Patel et al., 2022). Gender-specific differences were also observed: M. pneumoniae infections occurred more frequently in men, which is consistent with studies reporting greater susceptibility of men to lower respiratory tract infections and may reflect both immunological and behavioural differences (Waites and Talkington, 2004; Ngo et al., 2014; Klein and Flanagan, 2016; Waites et al., 2017). In contrast, other respiratory pathogens showed no consistent gender disparity. These findings highlight the importance of targeted surveillance and tailored prevention strategies for paediatric high-risk groups, including consideration of host factors such as age and gender in the post-COVID era.

The different distribution of respiratory pathogens in hospitalised and outpatient children underlines their different correlation with the severity of the disease. Pathogens such as *M. pneumonia*e, RSV, IFV-B and PIV-III are more frequently detected in hospitalised patients, probably reflecting their link with severe lower respiratory tract infections such as pneumonia and bronchiolitis, which often require hospitalisation (Liu et al., 2013). Conversely, ADV, IFV-A and PIV-I predominate in outpatients, suggesting milder clinical presentations that are manageable by outpatient treatment (Liu et al., 2013). These distribution patterns may be influenced in part by diagnostic practises—patients admitted to hospital tend to receive more extensive microbiological testing—as well as care-seeking behaviour, with more severe symptoms prompting hospital admission.

Our surveillance confirmed clear seasonal peaks for the most important respiratory pathogens in children. RSV and IFV-A showed a clear predominance in winter, consistent with patterns observed in temperate climates and long-term data from Hubei province (2014–2019) (He et al., 2023; Hu et al., 2023; Liu et al., 2025). These trends indicate a restoration of traditional winter seasonality post-COVID, which is consistent with reports from other regions of China. In contrast, AdV peaked in spring and early summer — an observation supported by some paediatric studies but not consistently seen in national-level data (Xu et al., 2021; He et al., 2023). As an unenveloped virus, AdV is known to circulate throughout the year, but its dominance in spring in Wuhan emphasises the importance of regional surveillance. This finding suggests that adenoviruses may become a major cause of respiratory infections in children after the winter virus season. This emphasises the need for local prevention strategies beyond the traditional flu season.

An important innovation of this study is the application of DLNMs to evaluate delayed meteorological effects on the transmission of respiratory pathogens. We found that low humidity significantly increased the risk of RSV infection after a delay of 1 to 2 weeks, suggesting that dry spells often precede an RSV increase in children. This finding is biologically plausible—cold, dry air increases the stability of enveloped viruses and impairs mucosal defences—and is consistent with previous studies from Edinburgh and nationwide Chinese data that identify humidity as a major factor in the seasonality of RSV in temperate regions (Price et al., 2019; Xu et al., 2021). By using the DLNMs, we were able to accurately determine the time window of peak susceptibility, a feature often overlooked by simple correlation methods. In contrast, the prevalence of adenovirus appeared to be largely unaffected by temperature or humidity, consistent with its year-round persistence and non-enveloped structure (He et al., 2023). IFV-A showed an intermediate pattern, with modest increases in cold, dry conditions. Overall, low humidity proved to be the most consistent climatic trigger for RSV transmission in Wuhan. While other trends may be observed in tropical areas, dryness appears to be the dominant environmental catalyst for RSV activity in temperate climates such as central China (Lee et al., 2023).

The differential effects of temperature and humidity on respiratory pathogen activity observed in our study can be largely attributed to variations in viral structure and environmental stability. Enveloped viruses, such as RSV and IFV-A, possess a lipid bilayer that is more stable in low-temperature and low-humidity conditions, likely due to reduced water activity that enhances viral survival in aerosols and on surfaces (Lowen et al., 2007). These conditions, prevalent in Wuhan’s winter months, facilitate prolonged viral viability and efficient transmission, explaining the pronounced winter peaks of RSV and IFV-A. Conversely, high temperatures or high humidity may disrupt the lipid envelope, leading to viral inactivation, which aligns with reduced activity of these pathogens in warmer, more humid seasons (Noti et al., 2013). In contrast, non-enveloped viruses like AdV have a robust protein capsid that confers greater resistance to environmental stressors, enabling survival across a wide range of temperatures and humidities (Stanford Environmental Health and Safety, 2025). This structural resilience explains AdV’s consistent circulation, particularly in spring and early summer in Wuhan, when enveloped viruses are less prevalent. Additionally, low humidity may impair host mucosal defenses, such as mucociliary clearance, increasing susceptibility to infections, particularly for RSV (Kudo et al., 2019). These pathogen-specific responses to climatic conditions underscore the importance of integrating virus ecology into predictive models for targeted public health interventions.

This study improves the understanding of paediatric respiratory infection patterns in the post-pandemic period. We provide timely evidence that Wuhan has resumed its seasonal rhythm from before the COVID pandemic, with RSV and IFV-A peaking in winter and AdV occupying a niche in spring/summer. This recovery is in line with global observations that seasonal epidemics re-emerge when immunity gaps are closed (Hu et al., 2023; Liu et al., 2025). An important innovation is our use of DLNMs to capture both immediate and delayed meteorological effects—for example, we found a one-week lag between low humidity and RSV increase. In contrast to conventional analyses based on monthly trends or direct correlations (Xu et al., 2021), this approach allows for precise quantification of temporal dynamics. To our knowledge, this is one of the first studies to apply DLNM modelling to paediatric respiratory viruses in central China. It provides valuable insights into how and when environmental factors influence pathogen activity.

These findings have direct implications for public health. The observed winter peaks of RSV and IFV-A suggest that preventive strategies — such as monoclonal RSV prophylaxis and influenza vaccination— should be implemented before the cold season (He et al., 2023). Conversely, the increase in AdV infections in spring suggests that clinicians should also be more vigilant about severe cases outside the traditional ‘flu season,” particularly in paediatric wards. Our data also highlight the potential of meteorological indicators — such as the drop in humidity— as an early warning system for RSV increases. Incorporating climate data into local forecasting models could enable timely interventions, including strengthening infection control measures or preparing hospital capacity. Finally, in the context of climate change, shifts in seasonal temperature and humidity could alter the timing and intensity of respiratory virus epidemics. These findings highlight the need for sustainable, climate-informed surveillance systems to support proactive control of paediatric infectious diseases.

To contextualize our findings, we compared them with studies from other regions in China and internationally that show consistent meteorological influences on respiratory infections in children that are modulated by regional climatic variations. In Suzhou, a subtropical region, Chen et al. (2014) eported RSV infections peaking in winter at low temperatures, which is consistent with our observations in Wuhan. However, our study uniquely highlights low relative humidity as a crucial factor for RSV transmission, an aspect that is less prominent in Suzhou. Similarly, in southern China, Cui et al. (2015) found that temperature and humidity influence respiratory virus activity, with AdV peaking in summer under warm, humid conditions, consistent with our AdV increase in spring in Wuhan, although regional climate differences influence seasonal timing. In Shaanxi’s temperate Shenmu county, Liu et al. (2016) identified temperature and humidity as key factors for pediatric lower respiratory tract infections, with RSV being predominant, confirming our findings. Sheffield and Landrigan (2011) demonstrated that climate change exacerbates respiratory infections and asthma in children through temperature variation and air pollution, especially during heat waves, which is consistent with the delayed meteorological effects we observed with GAMs. Mirsaeidi et al. (2016) also found an association between sudden drops in temperature and increased emergency visits for pneumonia in children, which is consistent with the lagged effects we found using the DLNM. These comparisons emphasize the need for region-specific, climate-based public health strategies to mitigate pathogen-specific risks for respiratory infections in children.

Extreme weather conditions were important factors in the dynamics of the pathogens, each of which had different risk profiles. High temperatures (~95th percentile, 33–34 °C) were associated with a notable increase in infection, particularly for RSV (RR ~1.6 at 33.8 °C), suggesting increased susceptibility to heat stress. In comparison, AdV, IFV-A and *M. pneumoniae* showed a lower increase (RR ~1.1–1.2). These findings are supported by studies linking heat exposure to systemic inflammation and suppressed antiviral immunity, which may increase susceptibility during heat waves (Lee and Yoon, 2024). In contrast, low temperatures (~5th percentile) in Wuhan had a milder effect, but are known to impair mucosal defences and promote respiratory diseases worldwide. Extreme dryness (35–45% RH) also increased the risk of RSV and AdV, consistent with previous findings that dry air promotes viral survival and impairs host defences (Lowen and Steel, 2014; Kudo et al., 2019). Conversely, IFV-A and *M. pneumoniae* peaked at high humidity (>80% RH), indicating different climate sensitivities. Such patterns are consistent with tropical epidemiology, where influenza often peaks during rainy, humid seasons (Lowen and Steel, 2014). Overall, these results emphasise that the responses of pathogens to climatic extremes are highly specific and that quantile-based models capture nuanced relationships between climateand infection that are often overlooked by average-effects approaches.

These results offer new epidemiological insights: Each climatic extreme — heat, cold or drought—selectively amplifies different respiratory pathogens, probably through different mechanisms such as viral stability, immune modulation and behavioural changes. Recognising these climate-specific susceptibilities enables targeted predictions and timely interventions. For example, increased RSV activity during heatwaves or a spike in AdV during droughts could serve as an early warning and allow healthcare systems to mobilise resources preventively. Given the growing interest in climate-informed disease surveillance, our results support the feasibility of integrating extreme weather forecasts into predictive models that support vaccination campaigns, public warnings or hospital preparedness (Morin et al., 2018). Importantly, the public health relevance of these patterns is amplified by climate change: with increasing heatwaves and shifting winters, traditional respiratory epidemic timing may be altered (Anderson et al., 2013). Our study highlights that the impact of climate extremes is pathogen-specific and actionable—underlining the need for adaptive, climate-resilient early warning systems tailored to local epidemiological contexts.

This study provides a comprehensive analysis of the post-pandemic dynamics of pediatric respiratory infections in Wuhan by integrating epidemiological patterns with demographic and meteorological data. Its major strengths include a large sample size, an extended surveillance period spanning multiple seasons, and the simultaneous analysis of seven major respiratory pathogens within a single pediatric cohort. The application of GAM and DLNM models enabled the detection of complex, nonlinear, and lagged associations between climate variables and infection rates, offering greater interpretive depth than conventional correlation methods. In addition, age- and setting-specific analyses allowed for a refined understanding of population susceptibility and seasonal burden.

However, several limitations should be acknowledged. First, this was a single-center study conducted at a pediatric tertiary hospital in Wuhan, which may limit the generalizability of findings to other settings with differing healthcare infrastructure or climate. Second, clinical severity indicators (e.g., ICU admission, oxygen therapy) were not available, precluding outcome-level analysis. Third, viral detection relied on qualitative PCR without subtyping or quantification, and bacterial co-infections were not systematically documented, potentially underestimating pathogen interactions. Fourth, IFV-B positivity was low with many null values, which may have affected model stability despite the use of negative binomial regression. Fifth, due to ethical and logistical constraints, structured residential data were not collected. As geographic information is classified as sensitive personal data, spatial analysis across Wuhan districts was not feasible in this single-center study. Future studies should expand to multi-center settings across different climatic regions to validate the observed climate–pathogen relationships. Integrating clinical severity indicators (e.g., ICU admission, respiratory support) and virological subtyping could help clarify links between environmental exposures and disease outcomes. Incorporating anonymized spatial data and host factors may further elucidate geographic and individual-level heterogeneity. Finally, interdisciplinary approaches combining meteorology, pediatrics, and data science are essential for developing climate-informed surveillance and early warning systems for pediatric respiratory infections.

Despite these limitations, our study offers several novel insights with practical implications for both epidemiological understanding and public health policy in China. We identified pathogen-specific climatic sensitivities—such as the strong association between low relative humidity and RSV, and the increased risk of AdV infections under warm and humid conditions—highlighting the critical role of climate–pathogen interactions. By applying DLNMs, we pinpointed lagged meteorological effects, revealing critical exposure windows that precede infection surges. These findings support the development of climate-informed early warning systems and the optimal timing of seasonal interventions such as RSV prophylaxis and influenza vaccination. Given China’s vast pediatric population and climatic diversity, incorporating meteorological indicators—such as humidity thresholds or temperature extremes—into routine respiratory virus surveillance could enhance epidemic preparedness. For example, a sustained drop in relative humidity could be used to trigger RSV alert levels in pediatric hospitals. Furthermore, the observed differences between inpatients and outpatients underscore the need for tiered clinical management strategies, such as prioritizing RSV prophylaxis for infants during dry winter months, and enhancing outpatient monitoring for AdV infections during spring. These practical measures could help optimize resource allocation, reduce hospitalization burden, and better protect vulnerable pediatric populations amid shifting environmental and immunity landscapes.

In conclusion, our findings highlight the value of incorporating climate variables in the prediction of respiratory disease and highlight the need for adaptive, pathogen-specific public health strategies to mitigate paediatric infectious disease risks amid ongoing climatic and epidemiological changes.

## Data Availability

The original contributions presented in the study are included in the article/**Supplementary Material**. Further inquiries can be directed to the corresponding authors.

## Supplementary material

Supplementary Figure 1 Three-dimensional lag–response surfaces from DLNMs illustrating associations between meteorological exposures and respiratory virus activity. Panels **(A–C)** present DLNM results for IFV-B, PIV-I, and PIV-III, respectively. IFV-B demonstrates immediate effects of temperature and delayed responses to wind speed. PIV-I exhibits rapid sensitivity to elevated temperature and humidity. PIV-III shows a delayed increase in risk under low humidity conditions, peaking at approximately lag day 21. These plots highlight the heterogeneous and nonlinear temporal relationships between meteorological variables and pathogen-specific infection risks.

Supplementary Figure 2 Quantile-based lag–response curves for extreme meteorological exposures among additional respiratory pathogens. Panels illustrate RR trajectories and 95% confidence intervals over lag periods of 0–21 days in response to the 5th (P5, red) and 95th (P95, blue) percentiles of temperature, relative humidity, and wind speed. Panels **(A–C)** correspond to IFV-B, PIV-I, and PIV-III, respectively. These findings underscore the heterogeneous and nonlinear effects of meteorological extremes on pathogen-specific transmission risks.

Supplementary Figure 3 Residual diagnostic plots for GAM models of respiratory pathogens. Each panel shows Pearson residuals versus fitted values (left) and normal Q–Q plots (right) for seven pathogens: *M. pneumoniae*, AdV, RSV, IFV-A, IFV-B, PIV-I, and PIV-III. The diagnostics indicate satisfactory model fit across most pathogens. Panels A–G correspond to MP, AdV, RSV, IFV-A, IFV-B, PIV-I, and PIV-III, respectively.
