# Supplementary figures and images for: Epidemiological trends and climatic drivers of pediatric respiratory infections in Wuhan, China: a multi-pathogen analysis
Source: Front Cell Infect Microbiol. 2025 Sep 4;15:1624638. doi: 10.3389/fcimb.2025.1624638 (PMC12443746; doi:10.3389/fcimb.2025.1624638)

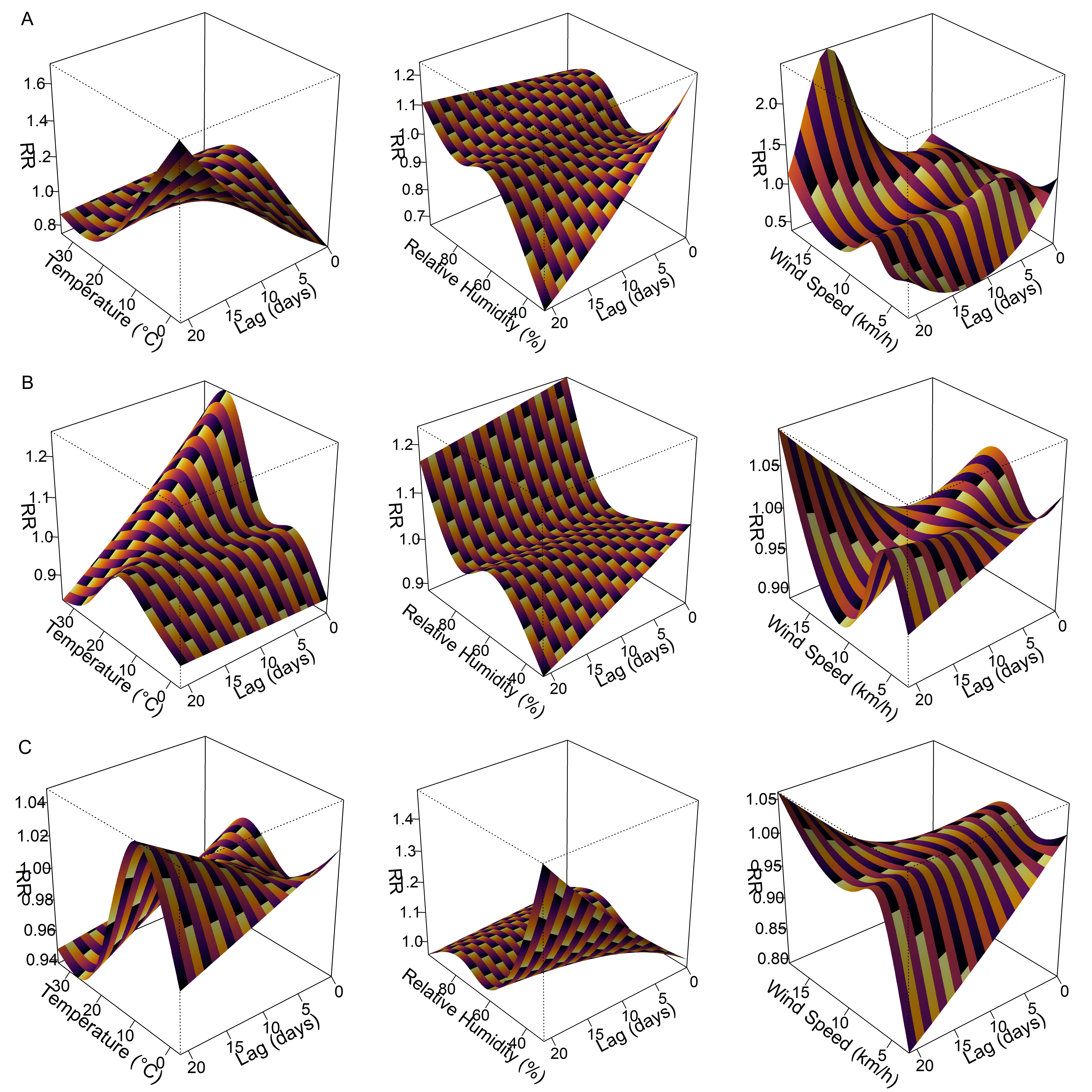

Supplement: Supplementary Figure 1 — Three-dimensional lag–response surfaces from DLNMs illustrating associations between meteorological exposures and respiratory virus activity. Panels (A–C) present DLNM results for IFV-B, PIV-I, and PIV-III, respectively. IFV-B demonstrates immediate effects of temperature and delayed responses to wind speed. PIV-I exhibits rapid sensitivity to elevated temperature and humidity. PIV-III shows a delayed increase in risk under low humidity conditions, peaking at approximately lag day 21. These plots highlight the heterogeneous and nonlinear temporal relationships between meteorological variables and pathogen-specific infection risks. [file Image1.jpeg]

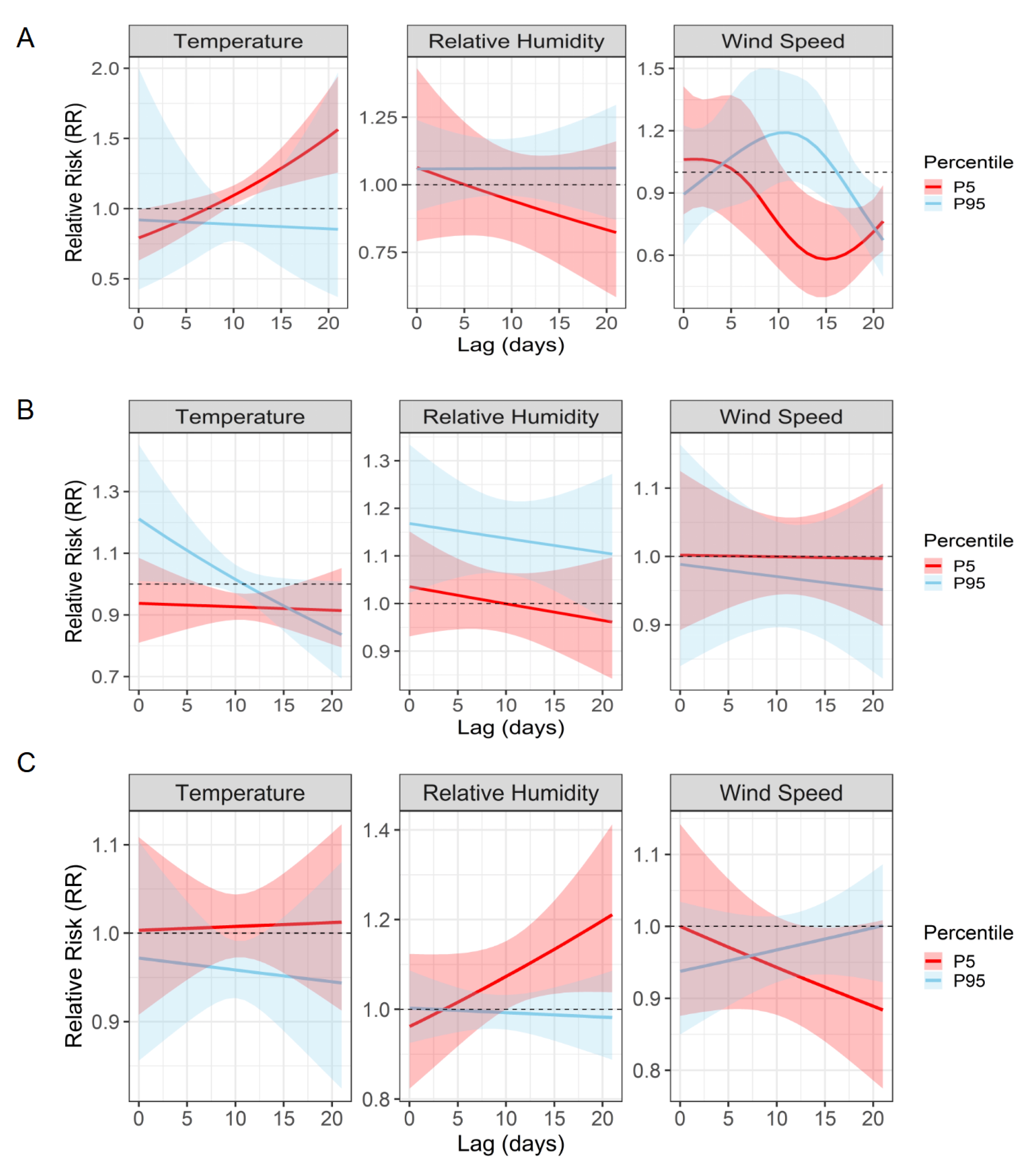

Supplement: Supplementary Figure 2 — Quantile-based lag–response curves for extreme meteorological exposures among additional respiratory pathogens. Panels illustrate RR trajectories and 95% confidence intervals over lag periods of 0–21 days in response to the 5th (P5, red) and 95th (P95, blue) percentiles of temperature, relative humidity, and wind speed. Panels (A–C) correspond to IFV-B, PIV-I, and PIV-III, respectively. These findings underscore the heterogeneous and nonlinear effects of meteorological extremes on pathogen-specific transmission risks. [file Image2.jpeg]

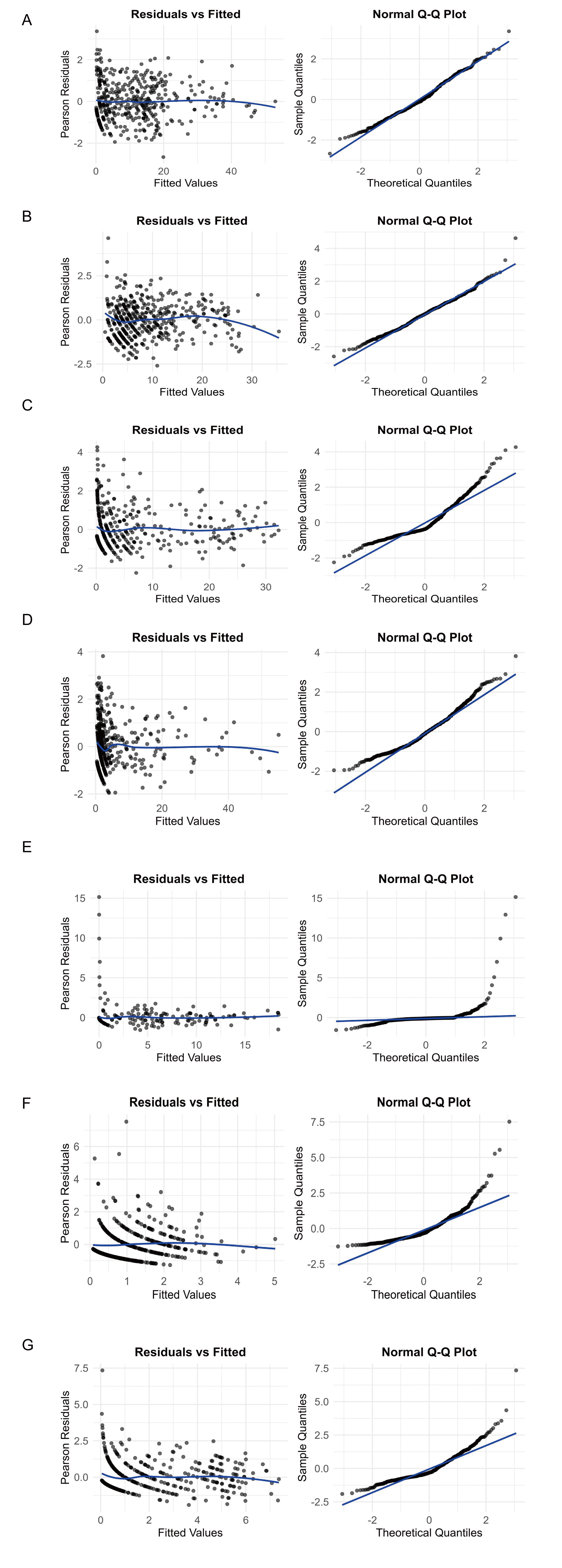

Supplement: Supplementary Figure 3 — Residual diagnostic plots for GAM models of respiratory pathogens. Each panel shows Pearson residuals versus fitted values (left) and normal Q–Q plots (right) for seven pathogens: M. pneumoniae, AdV, RSV, IFV-A, IFV-B, PIV-I, and PIV-III. The diagnostics indicate satisfactory model fit across most pathogens. Panels A–G correspond to MP, AdV, RSV, IFV-A, IFV-B, PIV-I, and PIV-III, respectively. [file Image3.jpeg]
