# Supplementary material for: Epidemiological trends and climatic drivers of pediatric respiratory infections in Wuhan, China: a multi-pathogen analysis
Source: Front Cell Infect Microbiol. 2025 Sep 4;15:1624638. doi: 10.3389/fcimb.2025.1624638 (PMC12443746; doi:10.3389/fcimb.2025.1624638)
Supplement: Supplementary file 4 [file Table1.docx]

# Supplementary Table: Pairwise Age Group Comparisons of Respiratory Pathogen Positivity Rates

| Pathogen | Age Group 1 | Age Group 2 | Bonferroni-adjusted p-value | Statistical Significance |
| --- | --- | --- | --- | --- |
| M. pneumoniae | <1 year | 1-3 years | <0.001 | Yes |
| M. pneumoniae | <1 year | 3-6 years | <0.001 | Yes |
| M. pneumoniae | <1 year | 6-11 years | <0.001 | Yes |
| M. pneumoniae | <1 year | >11 years | <0.001 | Yes |
| M. pneumoniae | 1-3 years | 3-6 years | <0.001 | Yes |
| M. pneumoniae | 1-3 years | 6-11 years | <0.001 | Yes |
| M. pneumoniae | 1-3 years | >11 years | <0.001 | Yes |
| M. pneumoniae | 3-6 years | 6-11 years | <0.001 | Yes |
| M. pneumoniae | 3-6 years | >11 years | 1.000 | No |
| M. pneumoniae | 6-11 years | >11 years | <0.001 | Yes |
| AdV | <1 year | 1-3 years | <0.001 | Yes |
| AdV | <1 year | 3-6 years | <0.001 | Yes |
| AdV | <1 year | 6-11 years | <0.001 | Yes |
| AdV | <1 year | >11 years | <0.001 | Yes |
| AdV | 1-3 years | 3-6 years | <0.001 | Yes |
| AdV | 1-3 years | 6-11 years | <0.001 | Yes |
| AdV | 1-3 years | >11 years | <0.001 | Yes |
| AdV | 3-6 years | 6-11 years | 0.0037 | Yes |
| AdV | 3-6 years | >11 years | <0.001 | Yes |
| AdV | 6-11 years | >11 years | <0.001 | Yes |
| RSV | <1 year | 1-3 years | <0.001 | Yes |
| RSV | <1 year | 3-6 years | <0.001 | Yes |
| RSV | <1 year | 6-11 years | <0.001 | Yes |
| RSV | <1 year | >11 years | <0.001 | Yes |
| RSV | 1-3 years | 3-6 years | <0.001 | Yes |
| RSV | 1-3 years | 6-11 years | <0.001 | Yes |
| RSV | 1-3 years | >11 years | <0.001 | Yes |
| RSV | 3-6 years | 6-11 years | <0.001 | Yes |
| RSV | 3-6 years | >11 years | <0.001 | Yes |
| RSV | 6-11 years | >11 years | 1.000 | No |
| IFV-A | <1 year | 1-3 years | <0.001 | Yes |
| IFV-A | <1 year | 3-6 years | <0.001 | Yes |
| IFV-A | <1 year | 6-11 years | 0.001 | Yes |
| IFV-A | <1 year | >11 years | <0.001 | Yes |
| IFV-A | 1-3 years | 3-6 years | 1.000 | No |
| IFV-A | 1-3 years | 6-11 years | 1.000 | No |
| IFV-A | 1-3 years | >11 years | 1.000 | No |
| IFV-A | 3-6 years | 6-11 years | 0.380 | No |
| IFV-A | 3-6 years | >11 years | 1.000 | No |
| IFV-A | 6-11 years | >11 years | 0.127 | No |
| IFV-B | <1 year | 1-3 years | 1.000 | No |
| IFV-B | <1 year | 3-6 years | 1.000 | No |
| IFV-B | <1 year | 6-11 years | <0.001 | Yes |
| IFV-B | <1 year | >11 years | 0.783 | No |
| IFV-B | 1-3 years | 3-6 years | 1.000 | No |
| IFV-B | 1-3 years | 6-11 years | <0.001 | Yes |
| IFV-B | 1-3 years | >11 years | 1.000 | No |
| IFV-B | 3-6 years | 6-11 years | <0.001 | Yes |
| IFV-B | 3-6 years | >11 years | 1.000 | No |
| IFV-B | 6-11 years | >11 years | 1.000 | No |
| PIV-I | <1 year | 1-3 years | 1.000 | No |
| PIV-I | <1 year | 3-6 years | 1.000 | No |
| PIV-I | <1 year | 6-11 years | 0.025 | Yes |
| PIV-I | <1 year | >11 years | 1.000 | No |
| PIV-I | 1-3 years | 3-6 years | 0.604 | No |
| PIV-I | 1-3 years | 6-11 years | <0.001 | Yes |
| PIV-I | 1-3 years | >11 years | 0.002 | Yes |
| PIV-I | 3-6 years | 6-11 years | <0.001 | Yes |
| PIV-I | 3-6 years | >11 years | 0.168 | No |
| PIV-I | 6-11 years | >11 years | 1.000 | No |
| PIV-III | <1 year | 1-3 years | 1.000 | No |
| PIV-III | <1 year | 3-6 years | <0.001 | Yes |
| PIV-III | <1 year | 6-11 years | <0.001 | Yes |
| PIV-III | <1 year | >11 years | <0.001 | Yes |
| PIV-III | 1-3 years | 3-6 years | <0.001 | Yes |
| PIV-III | 1-3 years | 6-11 years | <0.001 | Yes |
| PIV-III | 1-3 years | >11 years | <0.001 | Yes |
| PIV-III | 3-6 years | 6-11 years | <0.001 | Yes |
| PIV-III | 3-6 years | >11 years | 0.001 | Yes |
| PIV-III | 6-11 years | >11 years | 1.000 | No |
