# Supplementary material for: Epidemiological trends and climatic drivers of pediatric respiratory infections in Wuhan, China: a multi-pathogen analysis
Source: Front Cell Infect Microbiol. 2025 Sep 4;15:1624638. doi: 10.3389/fcimb.2025.1624638 (PMC12443746; doi:10.3389/fcimb.2025.1624638)
Supplement: Supplementary file 5 [file Table2.docx]

# Supplementary Table S2

Peak relative risks (RR), 95% confidence intervals (CI), and lag days for the associations between meteorological variables and respiratory pathogens, as estimated by distributed lag nonlinear models (DLNM). The table summarizes maximum effects extracted from the modeled exposure–lag–response surfaces.

| **Pathogen** | **Meteorological variable** | **Peak exposure value** | **Lag (days)** | **Peak RR** | **95% CI** |
| --- | --- | --- | --- | --- | --- |
| *M. pneumoniae* | Temperature | 33.8 °C | 0 | 1.09 | (1.01–1.17) |
| *M. pneumoniae* | Humidity | 97% | 18 | 1.06 | (1.00–1.12) |
| *M. pneumoniae* | Wind speed | 6.1 km/h | 11 | 1.01 | (1.00–1.02) |
| AdV | Temperature | 33.8 °C | 2 | 1.17 | (1.08–1.27) |
| AdV | Humidity | 44.3% | 0 | 1.16 | (1.05–1.28) |
| AdV | Wind speed | 5.4 km/h | 0 | 1.14 | (1.06–1.21) |
| RSV | Temperature | 33.8 °C | 21 | 1.61 | (1.22–2.12) |
| RSV | Humidity | 35.6% | 17 | 1.13 | (1.00–1.28) |
| RSV | Wind speed | 17.7 km/h | 0 | 1.32 | (1.05–1.66) |
| IFV-A | Temperature | 33.8 °C | 0 | 1.15 | (1.06–1.25) |
| IFV-A | Humidity | 84% | 12 | 1.27 | (1.14–1.40) |
| IFV-A | Wind speed | 3.8 km/h | 15 | 1.19 | (1.11–1.28) |
| IFV-B | Temperature | −2.7 °C | 21 | 1.70 | (1.31–2.21) |
| IFV-B | Humidity | – | - | - | - |
| IFV-B | Wind speed | 17.7 km/h | 15 | 2.47 | (1.21–5.04) |
| PIV-I | Temperature | 27.4 °C | 0 | 1.26 | (1.08–1.47) |
| PIV-I | Humidity | 97% | 0 | 1.24 | (1.02–1.50) |
| PIV-I | Wind speed | – | - | - | - |
| PIV-III | Temperature | 9.7 °C | 17 | 1.03 | (1.00–1.07) |
| PIV-III | Humidity | 31% | 21 | 1.49 | (1.08–2.05) |
| PIV-III | Wind speed | – | - | - | - |
